# Leveraging digital tools to enhance diversity and inclusion in clinical trial recruitment

**DOI:** 10.3389/fpubh.2024.1483367

**Published:** 2024-10-28

**Authors:** Tosin Tomiwa, Erin Wong, Hailey N. Miller, Oluwabunmi Ogungbe, Samuel Byiringiro, Timothy Plante, Cheryl R. Himmelfarb

**Affiliations:** ^1^Johns Hopkins University School of Nursing, Baltimore, MD, United States; ^2^Institute for Clinical and Translational Research, Johns Hopkins University School of Medicine, Baltimore, MD, United States; ^3^The Robert Larner, M.D. College of Medicine at The University of Vermont, Burlington, VT, United States; ^4^Johns Hopkins University Bloomberg School of Public Health, Baltimore, MD, United States

**Keywords:** social media, diverse population, digital methods, health equity, diversity in clinical research, inclusive recruitment, digital health

## Abstract

Clinical research is pivotal in assessing the safety and efficacy of new treatments in healthcare. However, the success of such research depends on the inclusion of a diverse and representative participant sample, which is currently lacking. This lack of diversity in biomedical research participants has significant repercussions, limiting the real-world applicability and accessibility of medical interventions, especially for underrepresented groups. Barriers to diverse participation include historical mistrust, logistical challenges, and financial constraints. Recent guidelines by government agencies and funding bodies emphasize the need for diversity in clinical trials, but specific strategies for inclusive recruitment are often lacking. This paper explores the use of digital methods to enhance diversity and inclusion in research recruitment. Digital tools, such as electronic medical records, social media, research registries, and mobile applications, offer promising opportunities for reaching diverse populations. Strategies include culturally tailored messaging, collaborations with community organizations, and the use of SEO to improve visibility and engagement. However, challenges such as privacy concerns, digital literacy gaps, and ethical considerations must be addressed. The promotion of diversity in clinical research recruitment is crucial for advancing health equity. By leveraging digital tools and adopting inclusive strategies, study teams can improve the diversity of study participants, ultimately leading to more applicable and equitable healthcare outcomes.

## 1 Introduction

Clinical research plays a crucial role in evaluating new treatments’ safety and efficacy in advanced healthcare. Their, particularly in later-phase studies assessing effectiveness, depends heavily on enrolling a diverse and representative sample of participants. The inclusion of participants with a wide spectrum of attributes, such as age, gender, ethnicity, nativity, and health conditions, is essential for fostering understanding and translating clinical research outcomes into real-world settings. However, the current state of participant recruitment in biomedical research shows a significant lack of diversity and inclusiveness (1). Addressing this issue is essential for achieving health equity. Employing innovative strategies to increase the inclusion of underrepresented populations in research can help address longstanding disparities, accelerate medical progress, and promote equitable health and inclusiveness (2).

While the issue of underrepresentation in health research has long been recognized, emerging studies reveal its far-reaching consequences. The lack of diverse participation in clinical trials limits the real-world effectiveness and accessibility of medical interventions for underrepresented groups (3). For instance, some novel findings have failed to provide the same benefits to individuals with diverse comorbidities or genetic variability not reflected in trial participants (4). The standard approach to cost-effectiveness analysis in health economics has been criticized for potentially perpetuating health disparities by focusing on short-term costs rather than long-term societal benefits (5). While addressing health disparities may require initial investments in additional research and potentially more costly treatments for underrepresented groups, evidence suggests that such investments can be cost-effective in the long term (6).

Reducing health disparities can lead to substantial economic benefits through improved productivity, reduced healthcare costs, and enhanced quality of life (7). Inadequate representation also contributes to higher rates of adverse events and reduced access for excluded groups, impacting the development of clinical guidelines and potentially exacerbating existing health inequities.

Several factors contribute to the underrepresentation of minority populations in clinical research. Mistrust in research, stemming from historical and ongoing unethical practices, is a significant barrier. Other barriers include safety concerns, fears of harm, time limitations, financial constraints, logistical difficulties, trust in the researcher and the reputation of institution, lack of access to information about research, among others (8).

Recent guidelines from government agencies, professional associations emphasize the importance of participant diversity in research (9). In the US, the National Institutes of Health now requires detailed plans for recruitment and enrollment. Also, the Food and Drug Administration, under the 2023 Consolidated Appropriations Act, mandates researchers and companies seeking approval for late-stage clinical trials to submit plans for ensuring diversity among participants (10). The National Institute for Health and Care Research (NIHR) in the United Kingdom has implemented the INCLUDE Ethnicity Framework to enhance minority inclusion (11) and the European Medicines Agency (EMA) emphasizes subgroup representation in trials.

While these guidelines mark progress, they lack specific guidance on inclusive recruitment strategies and best practices. At the same time, the growing complexity of research recruitment, driven by the surge of digital tools, increases the risk of exclusionary practices if these tools are used without considering diversity and inclusion. Thus, understanding and addressing barriers to diverse participation in recruitment and engagement approaches and leveraging digital tools is critical. In this paper, we provide an overview of digital methods applied to research recruitment and current guidance on best practices for leveraging digital tools and strategies to improve diversity and inclusion in the recruitment process for studies and research teams.

## 2 Promoting diversity, equity and inclusion in clinical trial recruitment

Recently, the importance of inclusivity in clinical research has been widely acknowledged. Despite progress in achieving gender balance, there remains a significant gap in representing underrepresented populations. Research teams still face challenges in recruiting individuals from racial and ethnic minorities, varied age groups, sexual orientations, gender identities, and those with disabilities (12).

The implications of this discrepancy extend far and wide. Most notably, the data derived from clinical research might lack applicability to a broader patient spectrum if diversity is not adequately addressed. Furthermore, the underrepresentation of specific cohorts in clinical studies perpetuates health disparities, limits our understanding of health intricacies, and hampers our ability to discern differences in treatment response among distinct demographic clusters (13). Moving forward, the establishment of a genuinely diverse patient population requires meticulous planning and a focus on patient-centric, culturally sensitive strategies. Achieving diversity, equity, and inclusion in clinical research recruitment necessitates integrating these principles throughout the research lifecycle, starting with the research team’s composition. Cultural competency training is essential to equip team members with an understanding of community dynamics, values, beliefs, and preferences, ensuring a transformative shift toward inclusive clinical research (14).

### 2.1 Research team expertise and diverse skill-sets

Lack of diversity among researchers and study teams perpetuates underrepresentation. Strategies to diversify the team include actively seeking collaborators, research partnerships, and mentees from underrepresented groups; recruiting through alumni networks, community and youth organizations, student associations, historically black colleges and universities, and within trusted spaces where people congregate (15). Mentorship is crucial for building a diverse research team; Research leaders dedicate time, effort, and resources into training students, trainees, and early-career professionals. Offering compensated research opportunities to students who might not otherwise have access can greatly enhance workforce diversity.

Skill diversity is also essential for engaging diverse populations, particularly when using digital methods. Technological, digital, and marketing literacy are increasingly vital for achieving equity in research. With rapid technological advancements, there is a need for updated digital skills, which are often missing from traditional research or clinical training. As digital tools become more prominent, it is imperative to include team members with expertise in digital technologies and marketing.

Several global initiatives have emerged to address these needs. For instance, the European Commission’s Marie Skłodowska-Curie Actions (MSCA) provide research fellowships that include mentorship and digital skills training, fostering diverse and interdisciplinary research teams (16). The African Academy of Sciences’ DELTAS Africa program offers mentorship and digital skills training for early-career researchers across Africa, showing promising results in improving research output and quality (17). In the US, the National Institutes of Health (NIH), through the Office of Clinical Research Training and Medical Education (OCRTME), offers a range of research and medical education training opportunities that encompass the entire career pathway spectrum, to cultivate a diverse, and innovative biomedical workforce (18). While these programs show potential in developing diverse, digitally skilled research teams, more comprehensive studies are needed to fully assess their long-term impact on research conduct and outcomes.

### 2.2 Identifying and reaching target audience

There are several digital platforms that can be used to identify and contact potential research participants. These include healthcare-related platforms, such as the electronic medical record (EMR) and associated patient portal, in addition to social media platforms and regional and national research registries. These digital recruitment methods have been major drivers of recruitment yields in recent years, facilitating reaching a large number of individuals in a timely manner (19). However, using these methods requires careful consideration to avoid inadvertently biasing research participant samples.

Usership rates for mobile devices and digital platforms are increasing across demographic groups; however, differences remain and must be considered in recruitment identification and outreach to avoid biasing participant samples. For instance, previous studies have reported recruiting a disproportionately non-Hispanic white participant sample when using patient portal messaging (20). This is likely due to the usership rates of the patient portal at a particular institution. However, strategies exist to mitigate this issue while still leveraging the strengths of the EMR for participant identification.

In a study recruiting adults with gout, researchers identified participants via the EMR and sent either a patient portal message or postal mail, based on their portal usage. They found that deploying both methods increased the recruitment of Black or African American adults. Other studies deploying multiple methods following EMR identification have also found differences in enrollment by sociodemographic characteristics, albeit varying by study (21). For example, in a study across five health systems, the EMR was used to identify and recruit bariatric surgery patients. Sites deployed various outreach methods following EMR identification, discovering that electronic strategies (email and patient portal) recruited a higher proportion of Black patients compared to in-person and postal mail (22). These findings emphasize the importance of considering usership rates when conducting recruitment to implement an inclusive strategy. They also highlight the strength of digital methodologies, which can leverage a targeted approach to reach underrepresented populations effectively.

Using digital methods allows for testing multiple versions of recruitment messages and employing an iterative approach to outreach. This method uses objective data to tailor messaging to diverse populations by customizing elements such as messaging verbiage, length, imagery, and outreach modality. Studies have shown that this approach can improve recruitment yields. For example, in the ADAPTABLE study (23), researchers compared click-through rates and enrollment outcomes between two Facebook ads, one culturally tailored and one not, among African American adults. The results showed higher click-per-impression ratios and enrollment yields with the tailored ads. Similarly (24), another study tested different subject lines, content, design, and cadence to optimize email-based recruitment, achieving a 12-fold increase from the initial approach to the optimized package.

### 2.3 Request/call for action

The success of digital recruitment in research relies on creating meaningful calls-to-action that prioritize the potential participant’s perspective and agency. This process involves three key steps: catching the individual’s attention, enabling informed action, and confirming the appropriate action was taken (25). While effective outreach is important, it is essential to balance recruitment efficiency with respect for participant autonomy and the principles of patient-centered research. The goal is not merely to generate “traffic,” but to connect individuals with research opportunities that may benefit them or contribute to scientific progress.

No matter the type of research study or the digital recruitment methods used, using well-crafted calls-to-action that resonate with the potential participant is vital for recruitment success. These calls provide clear guidance, allowing potential participants to take action, such as clicking to complete a short survey, sending an email, or making a phone call (26). It essential that the call-to-action stands out on the webpage and reflects participants priorities and motivations. Visually, this can be achieved by choosing a button color or linked text that contrasts with the rest of the page. Regardless of visualization, though, it is important that verbiage aligns with participants health goals or motivations to participate in research (27).

### 2.4 Search engine optimization

Utilizing search engine optimization (SEO) in clinical research recruitment can increase participant diversity by reaching a broader audience through inclusive search terms, metadata, and mobile optimization. SEO is a strategy that involves identifying the most popular search terms (keywords and phrases) for a selected topic and applying them to a recruitment advertisements or clinical research websites, to improve visibility in search results, thus increasing the likelihood that people will interact with them (28). For example, Hershberger et al. compared websites and other internet recruitment methods to traditional recruitment methods such as clinics and newsletters (29). They employed metadata and descriptions in their website code to help search engines categorize their site and support searchability, thus contributing to the recruitment of 82% of their study sample. The employment of metadata, or informational tags associated with a website, can be leveraged to increase digital content rankings and traffic (30) to support recruitment volume and diversity.

These search terms and metadata can be made more inclusive by tailoring language to be contextually appropriate for target audiences, taking accessibility needs into account, and inviting collaboration and feedback from community members to inform their development. Considerations for multilingual SEO may also be made to improve visibility. In addition, mobile optimization can boost user traffic with internet ads and websites by making a website and other digital content mobile-friendly. This is relevant to SEO because most internet searches are performed on mobile devices, and many major search engines have adopted mobile-first indexing, which favors websites oriented for mobile devices, regardless of what device used for the search (31). Understanding these broad industry practices, rather than focusing on any single platform, is relevant for researchers aiming to improve the visibility of their recruitment materials. Moreover, the use of search terms, metadata, and mobile optimization to support search discovery of digital content can also help with ADA compliance. There is an abundance of resources on implementing SEO in web-based materials, but there is a paucity of peer-reviewed literature on its application to clinical research recruitment and its relevance in meeting the needs of individuals with diverse backgrounds. The employment of SEO in developing digital recruitment materials is an opportunity to use language that is culturally inclusive, accessible, and resonates with diverse populations to encourage participation from communities that are underrepresented in clinical research (32).

In addition to encoding customized language into the infrastructure of web content with SEO, tailoring recruitment materials can help connect clinical research to broader audiences, with the goal of increasing representation and decreasing health disparities through clinical research opportunities. Several strategies may involve developing inclusive content, incorporating representative graphics and imagery, and engaging the community (33).

### 2.5 Tailoring

The development of inclusive content encompasses a broad range of considerations. One facet is the language itself, which includes person-first messaging, cultural relevance, accessibility (multilingual options, text-to-speech availability, etc.), level of readability, and avoiding exclusionary or biased language, to name a few. Another is whether the content is thematically welcoming to target audiences. Recruitment materials can highlight testimonials and experiences of diverse individuals, address health disparities, demonstrate a commitment to health equity, and be mindful of cultural stereotypes and other insensitivities (34). Barrera et al. synthesized and evaluated strategies for culturally adapting behavioral interventions to the populations they are intended for, emphasizing the benefits of employing culturally appropriate frameworks for more effective recruitment (35). Representative imagery through graphics, testimonials, and other methods in digital content can also contribute to engaging a wider audience and building trust with individuals. This visual storytelling could help individuals see themselves in the context of participating in clinical research and contributing to medical research in a way that transcends language. The American Chemical Society Guide to Scholarly Communication addresses inclusivity and representation in images. They provide a guide on using images to reflect one’s audience, emphasizing intentionality and respect in this process (36), as well as input from a diverse team (37). Mindfulness toward creating inclusive content is not a new idea, but doing so in a digital landscape provides the opportunity to leverage the vast network of platforms on the web to interact with more diverse audiences.

Finally, while not an exclusively digital strategy, investing in partnerships with communities of interest can help further tailor recruitment materials (38). These partnerships provide opportunities to participate in community events, build rapport, and collaborate with local leaders and organizations. Taking part in outreach offers face-to-face interactions and discussions to strengthen relationships between researchers and community members. Together, they can work together to develop recruitment materials, language options, best communication methods, and other elements that respect and support the unique needs and preferences of the community.

## 3 Challenges, opportunities and future directions

### 3.1 Challenges

Numerous challenges trail the use of digital methods in recruitment and in ensuring diversity in research recruitment. Some of these challenges include privacy concerns and confidentiality, internet accessibility, cost, information overload, and reduced quality of interaction (39). Ethical concerns with digital recruitment include issues such as consent, data security, algorithmic biases, and the digital divide. These issues are often overlooked by research stakeholders, posing challenges to ethical digital recruitment. Addressing this requires a multifaceted approach: training researchers and stakeholders, involving community representatives to ensure diverse perspectives, and conducting regular reviews to monitor security and accessibility. Collaborating with technology experts can improve user-friendly interfaces, encryption, and communication while promoting transparency and accountability. This approach ensures the ethical and effective use of digital recruitment strategies.

Cost and time are essential factors in research recruitment for sponsors, funders, institutions, and investigators. The use of digital tools for recruitment presents an ethical dilemma by potentially widening the digital divide and excluding populations with limited digital literacy. Broadband access issues exacerbate health and digital inequities. While digital tools like telehealth can improve access to care and research, they also leave behind individuals without access to devices, the internet, or technical skills. Additionally, research teams often lack the skills to adapt to and effectively use digital tools. Addressing these challenges requires strategic planning to ensure inclusivity and bridge the digital gap in research recruitment (40) and begs the question of the composition of research teams: should they comprise people with digital marketing expertise?

As digital recruitment strategies grow, many research sponsors are yet to incorporate guidance or funding for these efforts. For instance, the length of certain funding cycles limits researchers’ ability to develop effective digital and multipronged recruitment strategies. Often, there is limited funding to engage with diverse populations and hire diverse research staff. Oversight boards, like institutional review boards (IRBs), are only beginning to offer guidance on digital recruitment strategies, leaving many research teams without clear direction on initial steps and ethical boundaries. Clinical research operates within strict timelines and limited resources, often resulting in underfunded and understaffed recruitment efforts. The fast-paced digital landscape can make it challenging for researchers to implement best practices effectively. Digital inequities remain a significant challenge, potentially excluding individuals without digital access from research opportunities. Also, acquiring expertise in developing digital recruitment tools can be costly, and many research teams lack the funding to train staff in these skills. Addressing these challenges requires strategic planning and investment to ensure that digital recruitment is inclusive, ethical, and effective, ultimately bridging the digital divide and improving the diversity and reach of research participation.

### 3.2 Opportunities

Digital tools and strategies offer promising opportunities to increase diversity and representation in clinical research recruitment. Digital platforms transcend geographical limitations, allowing researchers to reach a wider and more diverse audience. However, their effectiveness varies depending on the nature of the trial. For predominantly virtual trials or those using Patient Reported Outcome Measures, digital tools can be particularly powerful. When in-person contact is required, these tools can enhance recruitment by effectively targeting local populations. This is especially relevant in multi-site and multi-country trials. Online advertisements, social media campaigns, emails, texts, etc. are cost-effective methods for mass recruitment and engagement tailored to specific demographics, but their use should be strategically aligned with the geographical scope and requirements of each individual study. Online advertisements, social media campaigns, emails, and text messages are just some methods of mass recruitment and engagement that can also be more cost-effective than print materials and in-person recruitment events (20, 41). Digital methods enhance access to research information, facilitate outreach, and encourage diverse participation in virtual recruitment events, webinars, and Q&A sessions. These events can be recorded, translated, transcribed, and distributed later. Online materials offer accessibility tools, including visual, auditory, and multilingual options, customizable to individual preferences. Digital recruitment methods can amplify representation and retention by effectively reaching target populations, especially when in-person recruitment is not feasible, such as during the COVID-19 pandemic. These strategies improve engagement and ensure that diverse groups have opportunities to participate in research and clinical studies (42–44). Digital efforts also contribute to more flexible communication, offering real-time interactions and tools to facilitate multilingual materials to decrease language barriers. Leveraging digital tools and strategies allows researchers unique opportunities to connect with more diverse audiences (45).

## 4 Conclusion

Promoting diversity in clinical research recruitment is essential for advancing health equity. A representative participant sample ensures that clinical research findings are applicable to real-world scenarios. Underrepresentation of certain demographic groups perpetuates health disparities and limits our understanding of effective solutions to health challenges. Addressing this requires a multifaceted approach, including diverse research teams and digital recruitment methods. Despite challenges and ethical considerations, digital tools offer significant opportunities to reach a wider and more diverse audience. They can overcome geographical barriers, provide cost-effective recruitment, and facilitate flexible communication with diverse populations.

Achieving health equity requires integrating diversity throughout clinical research, from team composition to recruitment methods. By addressing challenges and leveraging digital opportunities, researchers can enhance diversity and representation in recruitment, ultimately benefiting the broader patient population and advancing medical progress.

## Data Availability

The original contributions presented in the study are included in the article/Supplementary material, further inquiries can be directed to the corresponding author.

## References

[1] Health Equity Collaborative. Driving diversity and inclusion in healthcare research and access: the tools for dismantling structural and social inequities faced by underserved communities. (2021). Available from: https://healthequitycollaborative.org/white-paper-driving-diversity-and-inclusion-in-health-care-research-and-access/ (Accessed February 10, 2024).

[2] Eldridge L. What is the purpose of clinical trials?: The goals of different phases of clinical trials. (2022). Available from: https://www.verywellhealth.com/what-is-the-purpose-of-clinical-research-2249350. (Accessed February 10, 2024).

[3] National Academies of Sciences, Engineering, and Medicine, Policy and Global Affairs, Committee on Women in Science, Engineering, and Medicine, Committee on Improving the Representation of Women and Underrepresented Minorities in Clinical Trials and Research Bibbins-Domingo K Helman A, editors. Improving representation in clinical trials and research: Building research equity for women and underrepresented groups. Washington (DC): National Academies Press (US); (2022). Why diverse representation in clinical research matters and the current state of representation within the clinical research ecosystem. Available from: https://www.ncbi.nlm.nih.gov/books/NBK584396/. (Accessed February 10, 2024).

[4] Clark LT Watkins L Piña IL Elmer M Akinboboye O Gorham M. Increasing diversity in clinical trials: overcoming critical barriers. Curr Probl Cardiol. (2019) 44:148–72. doi: 10.1016/j.cpcardiol.2018.11.002, PMID: 30545650

[5] Brent RJ. Cost-benefit analysis versus cost-effectiveness analysis from a societal perspective in healthcare. Int J Environ Res Public Health. (2023) 20:4637. doi: 10.3390/ijerph20054637, PMID: 36901658 PMC10001534

[6] Nagy G Saldana L Gonzalez-Guarda RM. Cost—a hidden aspect of equity-grounded implementation science. JAMA health. Forum. (2024) 5:e242350. doi: 10.1001/jamahealthforum.2024.2350, PMID: 38900417

[7] LaVeist TA Gaskin D Richard P. Estimating the economic burden of racial health inequalities in the United States. Int J Health Serv. (2011) 41:231–8. doi: 10.2190/HS.41.2.c 21563622

[8] George S Duran N Norris K. A systematic review of barriers and facilitators to minority research participation among African Americans, Latinos, Asian Americans, and Pacific islanders. Am J Public Health. (2014) 104:e16–31. doi: 10.2105/AJPH.2013.301706, PMID: 24328648 PMC3935672

[9] Congress.gov. Text - H.R.7667 - 117th Congress (2021-2022): Food and Drug Amendments of 2022. (2022). Available from: https://www.congress.gov/bill/117th-congress/house-bill/7667/text. (Accessed March 12, 2024).

[10] Peloquin D Barnes M Lam C. Congress enacts legislation requiring guidance on clinical research diversity and modernization. (2023). Available from: https://www.ropesgray.com/en/insights/alerts/2023/01/congress-enacts-legislation-requiring-guidance-on-clinical-research-diversity-and-modernization. (Accessed March 12, 2024).

[11] NIHR. Improving inclusion of under-served groups in clinical research: Guidance from the NIHR-INCLUDE project. NIHR: UK (2020).

[12] Versavel S Subasinghe A Johnson K Golonski N Muhlhausen J Perry P. Diversity, equity, and inclusion in clinical trials: a practical guide from the perspective of a trial sponsor. Contemp Clin Trials. (2023) 126:107092. doi: 10.1016/j.cct.2023.107092, PMID: 36702295

[13] Konkel L. Racial and ethnic disparities in research studies: the challenge of creating more diverse cohorts. Environ Health Perspect. (2015) 123:A297–302. doi: 10.1289/ehp.123-A297, PMID: 26625444 PMC4670264

[14] Stubbe DE. Practicing cultural competence and cultural humility in the care of diverse patients. Focus (Am Psychiatr Publ). (2020) 18:49–51. doi: 10.1176/appi.focus.20190041, PMID: 32047398 PMC7011228

[15] Ruzycki SM Ahmed SB. Equity, diversity and inclusion are foundational research skills. Nat Hum Behav. (2022) 6:910–2. doi: 10.1038/s41562-022-01406-7, PMID: 35768648 PMC9243902

[16] European Commission. About Marie Skłodowska-curie actions. (2024). Available from: https://marie-sklodowska-curie-actions.ec.europa.eu/about-msca?pk_source=website&pk_medium=link&pk_campaign=hp&pk_content=hp-hero-discover. (Accessed September 28, 2024).

[17] African Academy of Sciences. Mentorship programme: focus areas. (2024). Available from: https://mentorship.aasciences.africa/focus-areas. (Accessed September 29, 2024).

[18] National Institutes of Health. Clinical Center training programs: office of clinical research training and medical education. (2024). Available from: https://www.cc.nih.gov/training. (Accessed September 29, 2024).

[19] Frampton GK Shepherd J Pickett K Griffiths G Wyatt JC. Digital tools for the recruitment and retention of participants in randomised controlled trials: a systematic map. Trials. (2020) 21:478. doi: 10.1186/s13063-020-04358-3, PMID: 32498690 PMC7273688

[20] Miller HN Charleston J Wu B Gleason K White K Dennison Himmelfarb CR. Use of electronic recruitment methods in a clinical trial of adults with gout. Clin Trials. (2021) 18:92–103. doi: 10.1177/1740774520956969, PMID: 32933342 PMC7878277

[21] Miller HN Berger MB Askew S Kay MC Chisholm M Sirdeshmukh G. Recruitment of diverse community health center patients in a pragmatic weight gain prevention trial. J Clin Transl Sci. (2023) 7:e22. doi: 10.1017/cts.2022.475, PMID: 36755547 PMC9879902

[22] Bennett W Bramante C Rothenberger S Kraschnewski JL Herring SJ Lent MR. Patient recruitment into a multicenter clinical cohort linking electronic health records from 5 health systems: cross-sectional analysis. J Med Internet Res. (2021) 23:e24003. doi: 10.2196/24003, PMID: 34042604 PMC8193474

[23] Cunningham-Erves J Kusnoor SV Villalta-Gil V Stallings SC Ichimura JS Israel TL. Development and pilot implementation of guidelines for culturally tailored research recruitment materials for African Americans and Latinos. BMC Med Res Methodol. (2022) 22:248. doi: 10.1186/s12874-022-01724-4, PMID: 36153481 PMC9508728

[24] Baca-Motes K Edwards AM Waalen J Edmonds S Mehta RR Ariniello L. Digital recruitment and enrollment in a remote nationwide trial of screening for undiagnosed atrial fibrillation: lessons from the randomized, controlled mSToPS trial. Contemp Clin Trials Commun. (2019) 14:100318. doi: 10.1016/j.conctc.2019.100318, PMID: 30656241 PMC6329362

[25] Mejtoft T Hedlund J Söderström U Norberg O. “Designing call to action: users’ perception of different characteristics.” In: *34th bled Econference: Digital support from crisis to progressive change: Conference proceedings*. University of Maribor University Press; (2021). p. 405–416.

[26] Martin K. Tips on creating compelling call-to-actions (CTAs) that call for action in healthcare settings. (2019). Available from: https://www.medtextpert.com/creating-compelling-call-to-actions-that-call-for-action-in-healthcare/#:~:text=Be%20concise.&text=Presented%20with%20too%20many%20options,now%27%20or2027today (Accessed March 18, 2024).

[27] Zillas. The psychology behind effective call-to-action buttons. (2024). Available from: https://www.designzillas.com/blog/psychology-behind-effective-call-action-buttons/. (Accessed September 28, 2024).

[28] Egri G Bayrak C. The role of search engine optimization on keeping the user on the site. Procedia Comput Sci. (2014) 36:335–42. doi: 10.1016/j.procs.2014.09.102

[29] Hershberger PE Kavanaugh K Hamilton R Klock SC Merry L Olshansky E. Development of an informational web site for recruiting research participants: process, implementation, and evaluation. Comput Inform Nurs. (2021) 29:544–53. doi: 10.1097/NCN.0b013e318224b52f, PMID: 21709545 PMC3572203

[30] An S Jung JJ. A heuristic approach on metadata recommendation for search engine optimization. Concurr Comput Pract Exp. (2019) 33:e5407. doi: 10.1002/cpe.5407

[31] Google Search Central. Mobile site and mobile-first indexing best practices. (2024). Available from: https://developers.google.com/search/docs/crawling-indexing/mobile/mobile-sites-mobile-first-indexing. (Accessed March 12, 2024).

[32] Google Search Central. Get started with search. (2024). Available from: https://developers.google.com/search/docs/fundamentals/get-started. (Accessed March 12, 2024).

[33] Mayers SA Cook SK Rantala C Israel T Helmer T Schorr M. The RIC recruitment & retention materials toolkit—a resource for developing community-informed study materials. J Clin Transl Sci. (2023) 7:e182. doi: 10.1017/cts.2023.607, PMID: 37706001 PMC10495822

[34] Thakur N Lovinsky-Desir S Appell D Bime C Castro L Celedón JC. Enhancing recruitment and retention of minority populations for clinical research in pulmonary, critical care, and sleep medicine: an official American Thoracic Society research statement. Am J Respir Crit Care Med. (2021) 204:e26–50. doi: 10.1164/rccm.202105-1210ST, PMID: 34347574 PMC8513588

[35] Barrera M Jr Castro FG Strycker LA Toobert DJ. Cultural adaptations of behavioral health interventions: a progress report. J Consult Clin Psychol. (2013) 81:196–205. doi: 10.1037/a0027085, PMID: 22289132 PMC3965302

[36] Ashwell SJ. ACS inclusivity style guide: diversity and inclusion in images. (2020). Available at: https://pubs.acs.org/doi/full/10.1021/acsguide.60107 (Accessed March 12, 2024).

[37] Ashwell SJ Fox-Morone MI. ACS inclusivity style guide: general guidelines. (2020). Available at: https://pubs.acs.org/doi/full/10.1021/acsguide.60102 (Accessed March 12, 2024).

[38] Puhan MA Steinemann N Kamm CP Mller S Kuhle J Kurmann R. A digitally facilitated citizen-science driven approach accelerates participant recruitment and increases study population diversity. Swiss Med Wkly. (2018) 148:w14623. doi: 10.4414/smw.2018.14623, PMID: 29767828

[39] Darko EM Kleib M Olson J. Social media use for research participant recruitment: integrative literature review. J Med Internet Res. (2022) 24:e38015. doi: 10.2196/38015, PMID: 35925655 PMC9389385

[40] Gleason KT Ford DE Gumas D Woods B Appel L Murray P. Development and preliminary evaluation of a patient portal messaging for research recruitment service. J Clin Transl Sci. (2018) 2:53–6. doi: 10.1017/cts.2018.10, PMID: 31660218 PMC6799557

[41] Brøgger-Mikkelsen M Ali Z Zibert JR Andersen AD Thomsen SF. Online patient recruitment in clinical trials: systematic review and meta-analysis. J Med Internet Res. (2020) 22:e22179. doi: 10.2196/22179, PMID: 33146627 PMC7673977

[42] Kelsey MD Patrick-Lake B Abdulai R Broedl UC Brown A Cohn E. Inclusion and diversity in clinical trials: actionable steps to drive lasting change. Contemp Clin Trials. (2022) 116:106740. doi: 10.1016/j.cct.2022.106740, PMID: 35364292 PMC9133187

[43] Lunn MR Lubensky M Hunt C Flentje A Capriotti MR Sooksaman C. A digital health research platform for community engagement, recruitment, and retention of sexual and gender minority adults in a national longitudinal cohort study—the PRIDE study. J Am Med Inform Assoc. (2019) 26:737–48. doi: 10.1093/jamia/ocz082, PMID: 31162545 PMC6696499

[44] Brewer LC Fortuna KL Jones C. Back to the future: achieving health equity through health informatics and digital health. JMIR Mhealth Uhealth. (2020) 8:e14512. doi: 10.2196/14512, PMID: 31934874 PMC6996775

[45] Chen J Wang Y. Social media use for health purposes: systematic review. J Med Internet Res. (2021) 23:e17917. doi: 10.2196/17917, PMID: 33978589 PMC8156131

