# Corrigendum: Leveraging digital tools to enhance diversity and inclusion in clinical trial recruitment

**DOI:** 10.3389/fpubh.2025.1613190

**Published:** 2025-04-29

**Authors:** Tosin Tomiwa, Erin Wong, Hailey N. Miller, Oluwabunmi Ogungbe, Samuel Byiringiro, Timothy Plante, Cheryl R. Himmelfarb

**Affiliations:** ^1^Johns Hopkins University School of Nursing, Baltimore, MD, United States; ^2^Institute for Clinical and Translational Research, Johns Hopkins University School of Medicine, Baltimore, MD, United States; ^3^The Robert Larner, M.D. College of Medicine at The University of Vermont, Burlington, VT, United States; ^4^Johns Hopkins University Bloomberg School of Public Health, Baltimore, MD, United States

**Keywords:** social media, diverse population, digital methods, health equity, diversity in clinical research, inclusive recruitment, digital health

In the published article, there was an error in the Funding statement. The NIH grant number was incorrect and a disclaimer was missing.

The correct Funding statement appears below.

