# Supplementary material for: Leveraging digital tools to enhance diversity and inclusion in clinical trial recruitment
Source: Front Public Health. 2024 Oct 28;12:1483367. doi: 10.3389/fpubh.2024.1483367 (PMC11550947; doi:10.3389/fpubh.2024.1483367)
Supplement: Supplementary file 1 [file Data_Sheet_1.PDF]

## 1 Supplementary Material

| Digital Methods for Research Recruitment | Strategies for Increasing Diversity and Inclusion |
|---|---|
| Electronic medical record and patient portals | Implement additional outreach methods and consider refining the query to give priority to underrepresented populations. |
| Social media advertising | Establishing collaborations with institutions and groups focused on underrepresented populations. |
| Regional and national research registries | Assess and evaluate the user rates of various recruitment channels and registries before initiating recruitment. |
| Web-based surveys and questionnaires | Enhance diversity by incorporating inclusive language and diverse visuals, using call-to-actions designed for the target audience, and ensuring that participants are engaged. |
| Search engine optimization | Culturally tailored messaging and materials. |
| Targeted online advertisements | Collaborating with community organizations and groups popular among underrepresented populations while ensuring that the ads are designed to be culturally relevant to encourage engagement. |
| Mobile applications | Engaging with community leaders for advocacy. |
| Email campaigns | Providing language interpretation services |
| Virtual focus group discussions | Hosting informational webinars or workshops |
